# Concurrent perception of competing predictions: A “split-stimulus effect”

**DOI:** 10.1167/jov.24.11.5

**Published:** 2024-10-08

**Authors:** Joseph Melling, William Turner, Hinze Hogendoorn

**Affiliations:** 1 Melbourne School of Psychological Sciences, the University of Melbourne, Melbourne, Australia; 2 Monash Centre for Consciousness and Contemplative Studies, Monash University, Melbourne, Australia; 3 School of Psychology and Counselling, Queensland University of Technology, Brisbane, Australia

**Keywords:** visual perception, predictive processing, motion illusion, consciousness, hyperpriors

## Abstract

Visual illusions are systematic misperceptions that can help us glean the heuristics with which the brain constructs visual experience. In a recently discovered visual illusion (the “frame effect”), it has been shown that flashing a stimulus inside of a moving frame produces a large misperception of that stimulus's position. Across two experiments, we investigated a novel illusion (the “split stimulus effect”) where the symmetrical motion of two overlaid frames produces two simultaneous positional misperceptions of a single stimulus. That is, one stimulus is presented but two are perceived. In both experiments, a single red dot was flashed when the moving frames reversed direction, and participants were asked to report how many dots they saw. Naïve participants sometimes reported seeing two dots when only one was presented, indicating spontaneous perception of the illusion. A Bayesian analysis of the population prevalence of this effect was conducted. The dependence of this effect on the frames’ speed, the dot's opacity, spatial attention, as the presence/absence of pre-flash motion (“postdiction”) was also investigated, and the features of this illusion were compared to similar motion position illusions within a predictive processing framework. In demonstrating this illusory “splitting” effect, this study is the first to show that it is possible to be simultaneously aware of two opposing perceptual predictions about a single object and provides evidence of the hyperpriors that limit and inform the structure of visual experience.

## Introduction

### Perception as inference

The empiricist philosopher John Locke was so certain that “[the senses] from external objects convey into the mind what produces there those perceptions,” that he grounded his entire epistemological theory upon its truth (Locke, 1690/1974). Research into the neural processing of sensory information, however, indicates that we should completely reverse this common-sense notion—perception is an unconscious inference that the brain makes about sensory inputs (Helmholtz, 1867/2005). This is because information “conveyed” into the mind by sensory organs is inherently indirect and ambiguous; they are the neural effects of sensory stimulation that lend themselves to multiple interpretations (Heeger, 2017). The brain must therefore make inferences about the “hidden” causal structure of the world, going beyond sensory data to do so (Hohwy, 2013; Rao & Ballard, 1999).

How is the brain able to go beyond its own inputs? It is commonly argued that the brain possesses and develops models that produce predictions of incoming sensory (e.g., Friston, 2005). Sensory inputs, then, function as feedback signals that show where these predictions err, allowing for model updating and more accurate predictions in the future. Importantly, this means representations in consciousness *are* perceptual inferences, not our sensory inputs. Locke's statement, then, can be amended: The mind from internal representations conveys to the senses what produces there those perceptions.

In this view, the function that the perceptual brain has evolved to perform is to infer the causes of changes in its sensory inputs (Friston, 2005; Gershman, Vul, & Tenenbaum, 2012; Rao & Ballard, 1999). To do this, the brain constructs models of the world that generate top-down predictions of information from the sense organs that are then compared to the actual bottom-up sensory inputs (Rao & Ballard, 1999). Where the inputs are accurately predicted, they are inhibited. This means the only bottom-up signal the brain uses is from unexpected inputs—a residual error signal that is used to update model parameters. Sensory inputs themselves, then, do not contain a “worldly state of affairs” that the brain is “passively soaking up in a bottom-up manner”; instead, the worldly state of affairs is contained in the perceptual predictions that inhibit expected sensory inputs (Hohwy, 2013).

The main function of perceptual processing can thus be understood as updating the parameters of the internal world models to minimize residual error. This process is thought to be essentially Bayesian (Friston, 2005; Heeger, 2017; Hohwy, 2013), with posterior hypotheses conceptualized as a set of model parameters calculated as a function of both the evidence from the senses and the models’ prior expectations. The upshot of this “predictive processing” account is that the content of perceptual consciousness depends on the set of model parameters that best minimize prediction error.

### Uncertainty and visual illusions

The predictive processing framework provides a natural account for what happens in contexts of sensory uncertainty, a major challenge for real-world perception. One way the brain can optimize the perceptual inferences it makes is by adjusting the gain of residual error signals based on estimates of their precision. Here, “gain” refers to the responsiveness of neurons or neural populations to potential sources of input and can be thought of as the weight given to sensory inputs in the perceptual inference process. For less variable sensory signals, which carry more precise information about the external state of the world, gain is increased. Conversely, for more variable signals, which carry less precise information, gain is reduced (Kwon, Tadin, & Knill, 2015).

One consequence of such precision-linked gain control is that in situations where uncertainty is high (e.g., when peripherally viewing a stimulus), prior expectations will be given relatively more weight in determining the consciously rendered perceptual account. For example, when a peripherally-viewed stationary object is filled with a moving texture, the perceived position of the object is shifted in the direction of the texture's motion (De Valois & De Valois, 1991; Ramachandran & Anstis, 1990). From this we can conclude that the brain integrates motion information into predictions about an object's position (Kwon et al., 2015). Crucially, the perceived positional shift scales with eccentricity due to the increased uncertainty that results from the decreased acuity of peripheral vision (see Anstis, 1998). That is, as the precision of sensory inputs decreases, the brain increasingly relies on its prior expectations from an internal model of motion dynamics (specifically, that motion leads to a change in position) when inferring the stimuli's true position (Kwon et al., 2015).

These priors can thus be conceptualized as biasing perception in contexts of uncertainty, and these biases can lead to the misrepresentations that characterize visual illusions. The study of visual illusions therefore provides a unique avenue to investigate how the brain generates perceptual prediction. By measuring the way in which the brain can *mis*represent certain artificial or uncertain stimuli, visual illusions allow us to reverse engineer the mechanisms the brain uses to represent the natural world under normal conditions (Grush, 2005).

### The present study: A new “split-stimulus effect”

In addition to providing a parsimonious account of various motion-position illusions, predictive processing also provides a natural explanation of effects like multistability (see Attneave, 1971). The brain can process competing hypotheses in parallel, and generally the hypothesis that pierces conscious awareness at any single time point is determined abductively—an inference to best explanation. This may lead one to wonder what happens if two or more competing hypotheses are deemed equally likely. These situations can give rise to multistability effects like binocular rivalry (see Alais, 2012), where instead of representing in consciousness an amalgam of both competing hypotheses, alternations between the competing percepts occur across time (Gershman et al., 2012). Hitherto, there is no evidence that two opposing hypotheses about a single object can pierce consciousness simultaneously.

**Figure 1.**
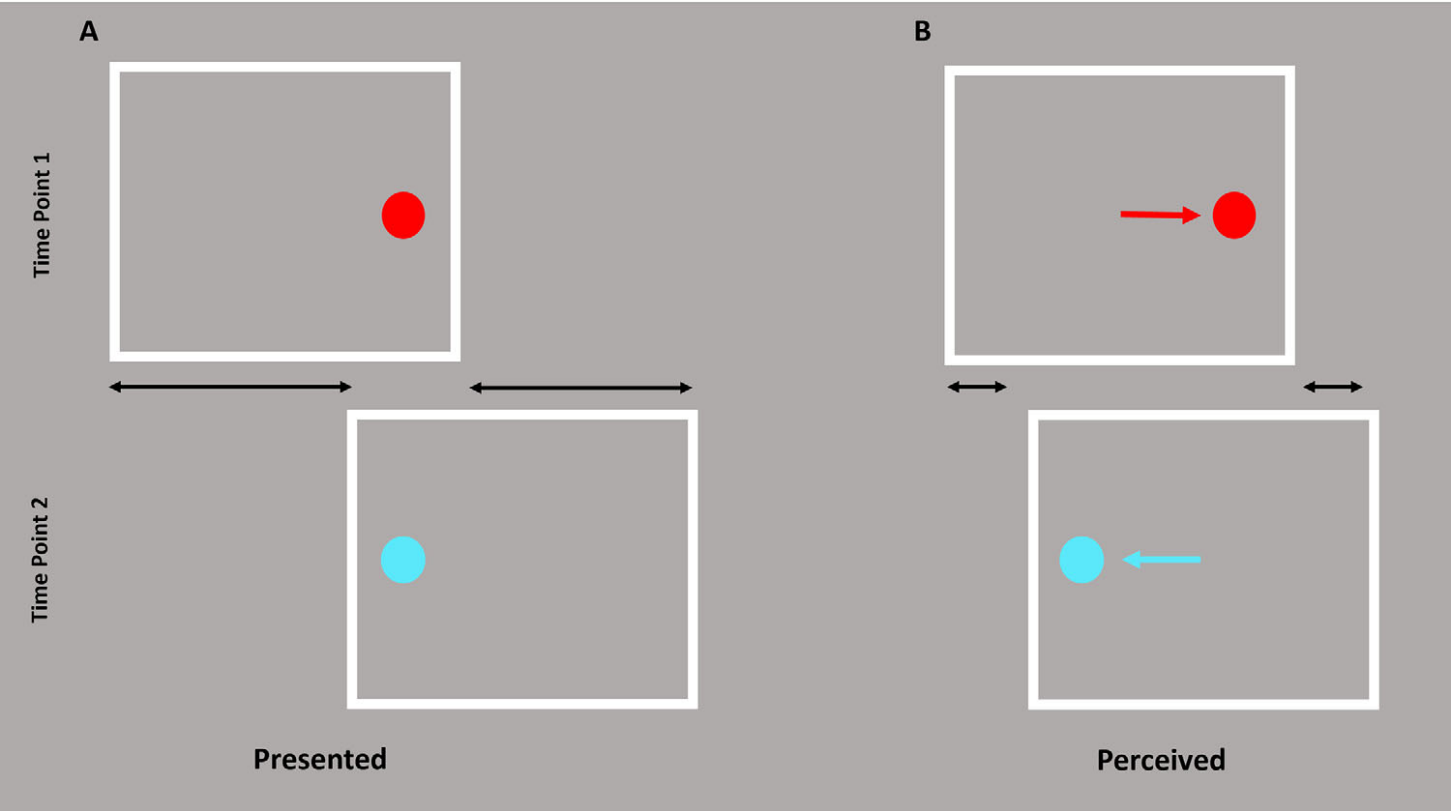
The frame effect. A single white frame oscillated laterally on a gray background, shown here at either end of its trajectory. (**A**) A red dot is flashed centrally when the frame pauses on the left, and a blue dot is flashed centrally when the frame pauses on the right. (**B**) Although the frame is perceived to be moving, its perceived trajectory is shortened (black arrows). The flashes are perceived to be shifted to their position relative to the frame (colored arrows) as though the frame were almost stationary. Adapted from “Paradoxical Stabilization of Relative Position in Moving Frames” by M. Özkan, S. Anstis, B. M. ’t Hart, M. Wexler, & P. Cavanagh, 2021, *Proceedings of the National Academy of Sciences*, 118(25) p. 3. Copyright © 2021 by PNAS.

Here we report a new “split-stimulus effect,” an adaptation of two recently reported illusions in which visual motion signals bias an object's perceived position: the frame effect (Özkan, Anstis, ’t Hart, Wexler, & Cavanagh, 2021) and the flash-grab effect (Cavanagh & Anstis, 2013). In the frame effect, the position of a flashed object is misperceived when presented within a moving frame. In Özkan et al.’s paradigm (2021), shown in Figure 1, a frame oscillates laterally with short pauses at either end of its path. While paused, a dot is flashed in the center of the path. Their findings indicate that the absolute location of the flashed stimulus was misperceived as the stimulus's relative position within the frame (i.e., if the stimulus appeared on the left-hand side of the frame when paused at the rightmost end of its trajectory, the dot would be misperceived as left-of-center). The confusion of the flash's perceived absolute position for its relative position indicates complete discounting of the frame's trajectory despite subjects reporting that they see such motion clearly.

The split-stimulus effect adapts the frame effect paradigm by presenting two overlaying semi-transparent frames that translate horizontally with opposite phase. To briefly foreshadow our findings, we demonstrate that a single dot flashed on the overlapping frames as they reverse direction is perceived as two separate dots, each shifted in the direction of the motion of one of the frames. Viewed through the lens of predictive processing, this indicates that two competing inferences about the object's position enter consciousness, such that it is perceived to “split” into two. This demonstrates for the first time that it is possible for two perceptual inferences about a single object to concurrently pierce conscious awareness. In two sets of experiments, we address two key empirical questions: (1) What is the prevalence of this novel effect in the naïve population, and (2) To what degree does this effect depend on factors such as frame speed, dot opacity, spatial attention, and the presence/absence of prior motion? In the following section, we briefly review these four factors of interest and outline how exploration of their influence may help to characterize the split-stimulus effect in relation to other potentially related illusions.

### Frame speed

Another potentially related visual illusion in which the position of a briefly flashed, stationary stimulus is misperceived due to a change in the direction of background motion is the Flash-Grab Effect (Cavanagh & Anstis, 2013). Building on the known phenomenon that the perceived trajectory of an object moving back and forth is shorter than its true trajectory (Sinico, Parovel, Casco, & Anstis, 2009), Cavanagh and Antis (2013) flashed a stimulus on an oscillating textured ring at the moment of motion reversal and found that the flash's position was misperceived at the same distance as the ring's perceived trajectory was shortened.

**Figure 2.**
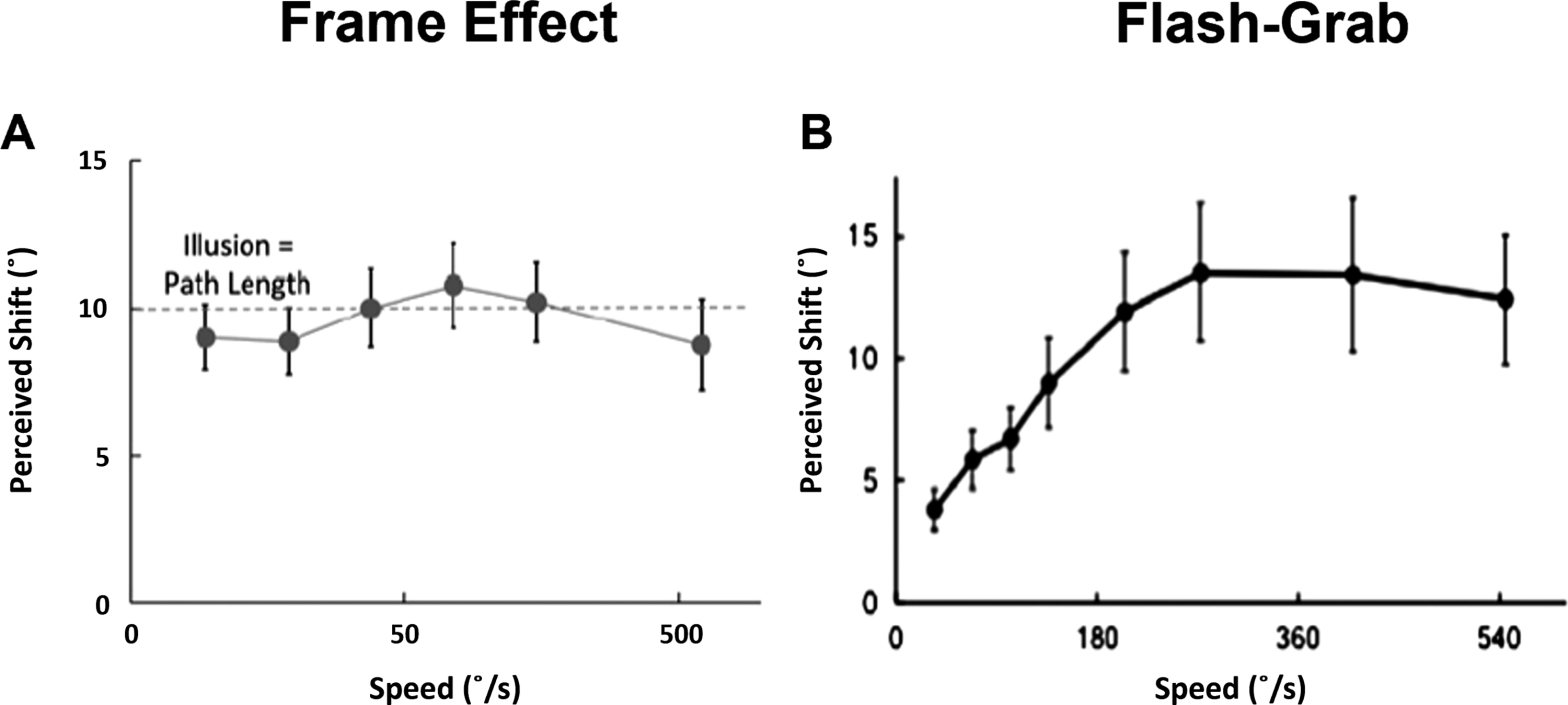
The frame effect and flash-grab effect as a function of speed and motion proximity. (**A**) The perceived shift of flashed stimuli in the frame effect as a function of the frame's speed. Speed of frame traversal, varying between 9.4°/s (traversals lasting 1066 ms) and 625°/s (traversals lasting 16 ms), had no significant effect of perceived shift. (**B**) The perceived shift of flashed stimuli in the flash-grab effect as a function of the surrounding ring's rotational speed. Perceived shift increases with speed before plateauing. Note the x-axis of (**A**) scales logarithmically. Also note the axis of (**A**) indicates lateral degrees of visual angle whereas the axis of (**B**) indicate radial degrees of visual angle. (**A**) adapted from “Paradoxical Stabilization of Relative Position in Moving Frames” by M. Özkan, S. Anstis, B. M. ’t Hart, M. Wexler, & P. Cavanagh, 2021, *Proceedings of the National Academy of Sciences*, 118(25) p. 4. Copyright © 2021 by PNAS. (**B**) adapted from “The Flash Grab Effect” by P. Cavanagh and S. Anstis, 2013, Vision Research, 91, p. 15. Copyright © 2013), with permission from Elsevier.

Given that the frame effect and the flash-grab effect both involve the shortening of a path trajectory and the illusory shifting of a flashed stimulus, one might imagine that these two phenomena result from similar mechanisms. Özkan et al. (2021), however, take pains to point out that despite the superficial resemblances between these effects, they differ in important ways. As shown in Figures 2A and 2B, speed of motion influences the perceived position shift of the flashed stimulus differently in the frame effect compared to the flash-grab effect. Of note, Özkan et al. (2021) found a near-constant illusory effect across frame traversal speeds (although they remark that there is no perceived shift when frame traversals reach the very slow speed of 1.5°/s). No conclusive explanation has yet been presented for the mechanism behind the rather large effect size of this illusion, nor for its lack of dependence on the speed of motion, but Özkan et al. (2021) speculate that the motion discounting demonstrated in the illusion may contribute to visual stability across saccades. In contrast to the frame effect, the flash-grab effect scales more or less linearly with speed up to a maximum of the perceived shift up to a maximum before plateauing, a pattern which is typical of other motion-position illusions (MPIs; e.g., Nakayama & Holcombe, 2021; Whitney & Cavanagh, 2000; Wojtach, Sung, Truong, & Purves, 2008).

With Experiment 1A, we measured the degree to which the split-stimulus effect is affected by the frames’ motion speed in the hopes of identifying shared mechanisms with either the frame effect or the flash-grab effect. If the size of position shift is a constant that arises once a certain threshold of motion speed is reached, as in the frame effect, then we expect to find no effect of increasing the speed of the frames’ motion on the likelihood of perceiving the illusion (except, perhaps, for a complete absence of perceiving the illusion at the slowest speeds). If the split-stimulus effect shares a mechanism with the flash-grab effect, however, the size of the position shifts should increase linearly with frame speed, which would in turn make the splitting easier to identify resulting in an increase to perceiving the illusion.

### Flash opacity

As mentioned above, a predictive processing interpretation of MPIs turns on an idea of precision weights. If incoming sensory information about the position of an object is imprecise, then motion information can be used to predict the object's present position. In Experiment 1B, the opacity of the flashed dot was varied in an attempt to manipulate the positional certainty of the flash. It was thought that decreasing the opacity of the flashed dot to lead to uncertainty about the flash's position (see Kanai, Sheth, & Shimojo, 2004), which would consequently lead to recruitment of motion information from the two frames. That is, we expect increased perception of the illusion as opacity decreases.

### Spatial attention

In Experiments 1A and 1B, participants knew with certainty the spatial location of the target flash; however, focused spatial attention has been shown to mediate the perceived position shift in the flash-grab effect (Adamian & Cavanagh, 2024), as well as related illusions like the “twinkle goes” effect (Nakayama & Holcombe, 2021).

In Experiment 2A, we investigated whether splitting attention between two locations in space would affect the size of the perceived position shift as in paradigms like Nakayama and Holcombe (2021). Specifically, a set of frames were presented both above and below fixation, creating two locations where the flashed target could appear. If a similar mechanism underlies the flash-grab effect and the split-stimulus effect, then we would expect an increased position shift when the flash's location is unknown and spatial attention must be split across both locations compared to when the flash's location is known and spatial attention could be focused to that location (Adamian & Cavanagh, 2024).

### Preceding motion

Many MPIs, including the Flash-Grab Effect, are primarily driven by motion that comes after the flashed target (Anstis & Cavanagh, 2017; Blom, Liang, Hogendoorn, 2019; Cavanagh & Anstis, 2013; Eagleman & Sejnowski, 2000; Takao, Sarodo, Anstis, Watanabe, & Cavanagh, 2022; van Heusden, Harris, Garrido, & Hogendoorn, 2019). In Experiment 2B, we investigated whether post-flash motion information is similarly sufficient for perceiving the Split-Stimulus Effect using a paradigm where no motion occurred before the appearance of the flashed target.

## Experiment 1

The aims of Experiment 1 were (1) to demonstrate the Split-Stimulus Effect in a sample of naïve observers, (2) to investigate the dependence of the effect on two key stimulus parameters (frame speed and flash opacity), and (3) to estimate the prevalence of the effect in the broader population.

### Methods

#### Participants

Forty-five participants (31 female) aged 18 to 33 years (*M* = 21.4, *SD* = 3.8) were recruited. Each participant gave written informed consent before beginning the experiment. All participants confirmed they had normal or corrected-to-normal vision, no history of neurological disorders, and at least two doses of a COVID-19 vaccine approved by the Therapeutic Goods Administration of Australia.

Participants were recruited using the Melbourne School of Psychological Sciences Reimbursed Participant System or the Melbourne School of Psychological Sciences Research Experience Program. Participants from the former system were reimbursed $10 at the end of the experiment; participants from the latter were given one research experience credit. The study was approved by the Human Ethics Committee of the Melbourne School of Psychological Sciences (ID 2022-12816-29275-8).

#### Apparatus

All experiments were conducted in a dimly lit room using a gamma-corrected 24.5-inch ASUS PG258Q monitor (ASUS, Taipei, Taiwan), with 1920 × 1080 resolution and 120 Hz refresh rate. The monitor was operated by an HP EliteDesk 800 G5 SFF (Hewlett-Packard Japan, Tokyo, Japan). Stimuli were made with the PsychoPy (v. 2022.1.1) package for Python (v.3.9.7) using the integrated development environment Spyder (v.5.1.5). Participants viewed the monitor from a chinrest at a distance of 50 cm.

#### Stimuli

**Figure 3.**
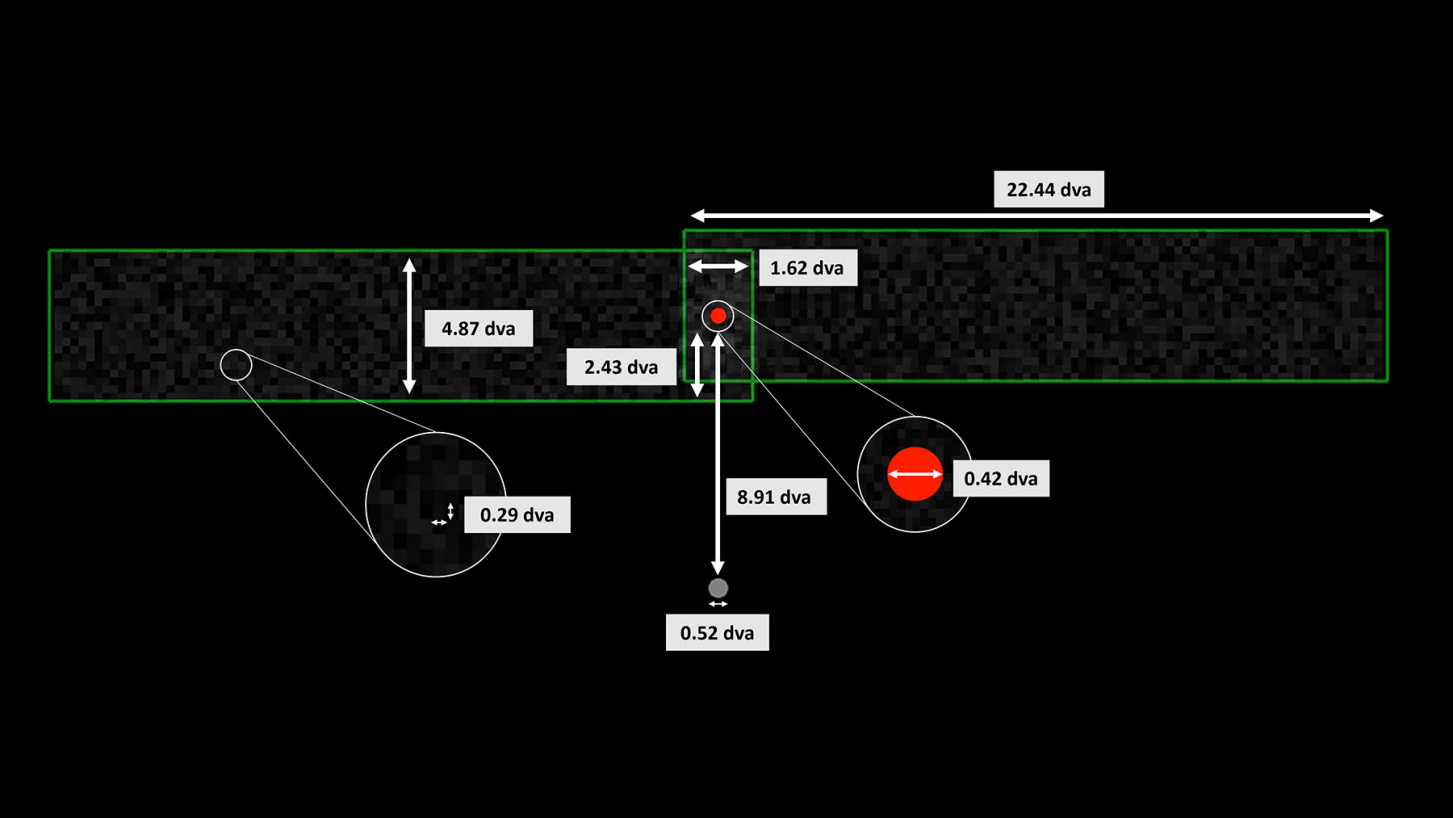
Stimulus dimensions during flash. Each frame moved symmetrically on a lateral path that was 5.84 dva in length in the speed block and 4.87 dva in the opacity block. Figure to scale, with the exception of the cutaways and the exact distance of the fixation dot from the stimulus.

Stimuli were presented on a uniformly black background. A gray fixation dot was presented at the center of the screen. Two overlaid green frames, filled with partially transparent uniformly-distributed luminance noise (opacity 8%), appeared at 8.91° of visual angle (dva) above fixation and translated horizontally back and forth with opposing phase. A red dot was flashed, centered on the overlapping region of the two frames at the moment that they reversed direction at the outermost point of their trajectory (see Figure 3 below). A video of the stimulus can be seen in Supplementary Movie 1.

#### Task

Participants reported the number of red dots that they saw on each trial using a computer keyboard. They had three response options corresponding to whether they saw zero, one, or two red dots on that trial. Participants were informed that each trial would continue until they made a response and were instructed to respond as soon as they had made a judgement. They were further informed that the number of dots flashed at frame reversal would be the same within each trial. They were also assured that it was ok if they used some responses more than others over the course of the experiment. Reporting that two dots were perceived in an experimental trial was interpreted as perceiving the illusion.

Catch trials made up 20% of total trials, evenly divided over the different experimental conditions. In catch trials, the red target dot was not presented. The purpose of catch trials was to estimate the base rate at which individual participants committed errors of commission (i.e., reported a dot when none was presented). Even in the absence of any illusion, participants might similarly make errors of commission and report two dots when only one was presented. Under the assumption that the error rate was constant across conditions, the error rate in catch trials therefore formed a baseline to which the rate at which participants reported perceiving two dots could be compared.

#### Experiment procedure

Participants were given both printed and verbal instructions. To avoid biasing participants’ reports, participants were told that the experiment investigated how motion in peripheral vision affects performance on a visual identification task. It was emphasized that maintaining fixation was important. They were otherwise not told the true purpose of the study until after the experiment, where they were informed of the illusion and asked about their experiences.

Experiment 1 manipulated two different stimulus parameters in separate blocks. In Experiment 1A, the speed of the moving frames was varied across trials between 3.9 °/s and 35 °/s in nine uniform steps. The opacity of the flashed dot was 100%. In Experiment 1B, the flashed dot's opacity varied across trials from 50% to 100% in six uniform steps. The speed of the frames was fixed at 23.4 °/s. The order of the two blocks was determined randomly. A screen before the first trial informed participants of which block they were about to complete.

Each participant completed 600 trials in total: 360 trials in Experiment 1A and 240 trials in Experiment 1B. Participants were presented with each of the nine frame speeds 40 times. Participants were presented with each of the six flash opacities 32 times. Trial order within blocks was randomized. Breaks were built in every 20 trials. All participants completed 20 practice trials with the option of completing more if they felt it necessary. Only one participant chose to do more than 20 practice trials, completing 40 in total. After the experiment, participants were debriefed using a short semi-structured interview. Questions about the nature of their percepts were asked before revealing that the experiment was attempting to induce a visual illusion. Debriefing interviews lasted approximately five minutes.

#### Trial procedure

**Figure 4.**
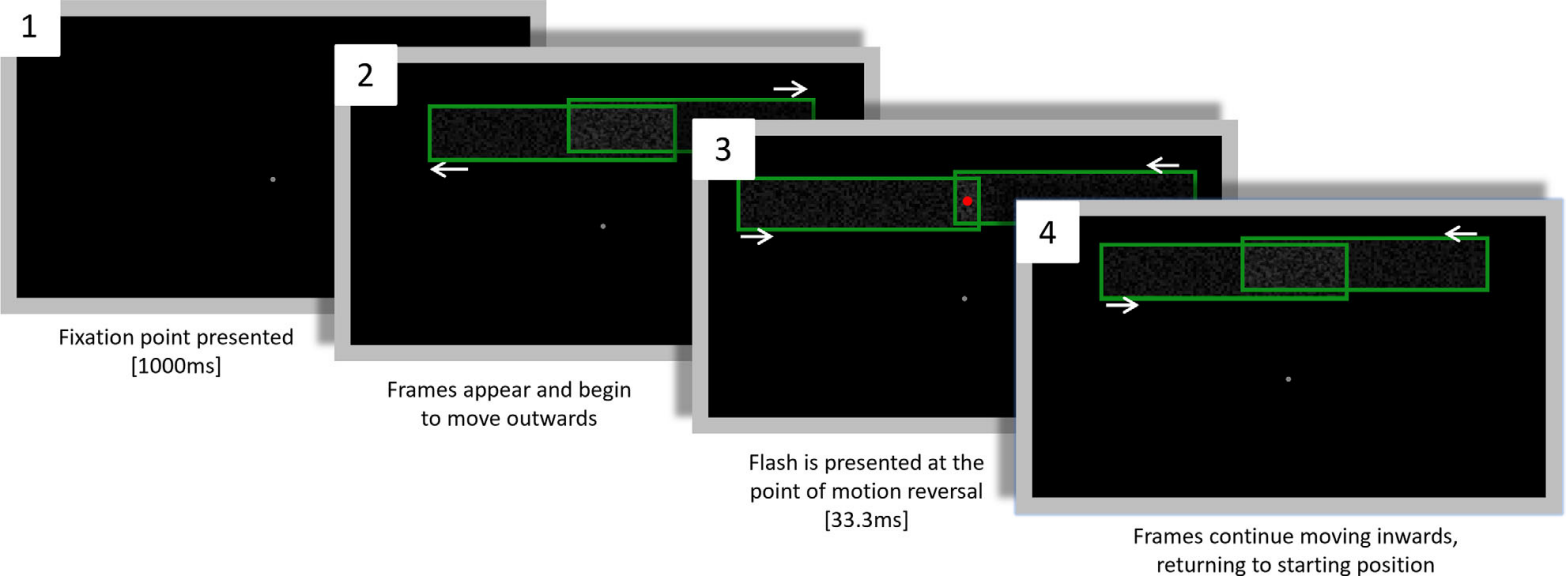
Trial procedure for Experiment 1. White arrows in (2, 3, and 4) indicate frame motion. (1) The fixation dot is presented on an otherwise blank screen. (2) Two frames appear and begin moving away from one another. (3) The frames reach the end of their trajectory and reverse direction. At the point of motion reversal a red dot is presented centrally (in non-catch trials) for 33.3 ms. (4) The frames continue to move towards one another until they return to the original position they appeared in (2). (2) to (4) then loop until participants respond. Participants could not respond until the first occurrence of (3). Figure not to scale.

The procedure for individual trials is shown in Figure 4. Each trial began with the presentation of a gray fixation dot for 1000 ms. The two overlapping green frames would then appear in motion, moving away from one another. When the frames reached the end of their trajectory, they would reverse direction, moving toward one another. A red dot was presented for 33 ms (four frames at 120 Hz) simultaneous with motion reversal. Participants could not make a response until after the first presentation of the red dot (or until the equivalent time point in catch trials). The frames’ oscillatory motion and the dot flashes continued until participants made a response. For description of analysis and code used, see: https://osf.io/b4m7d/**.**

### Results

**Figure 5.**
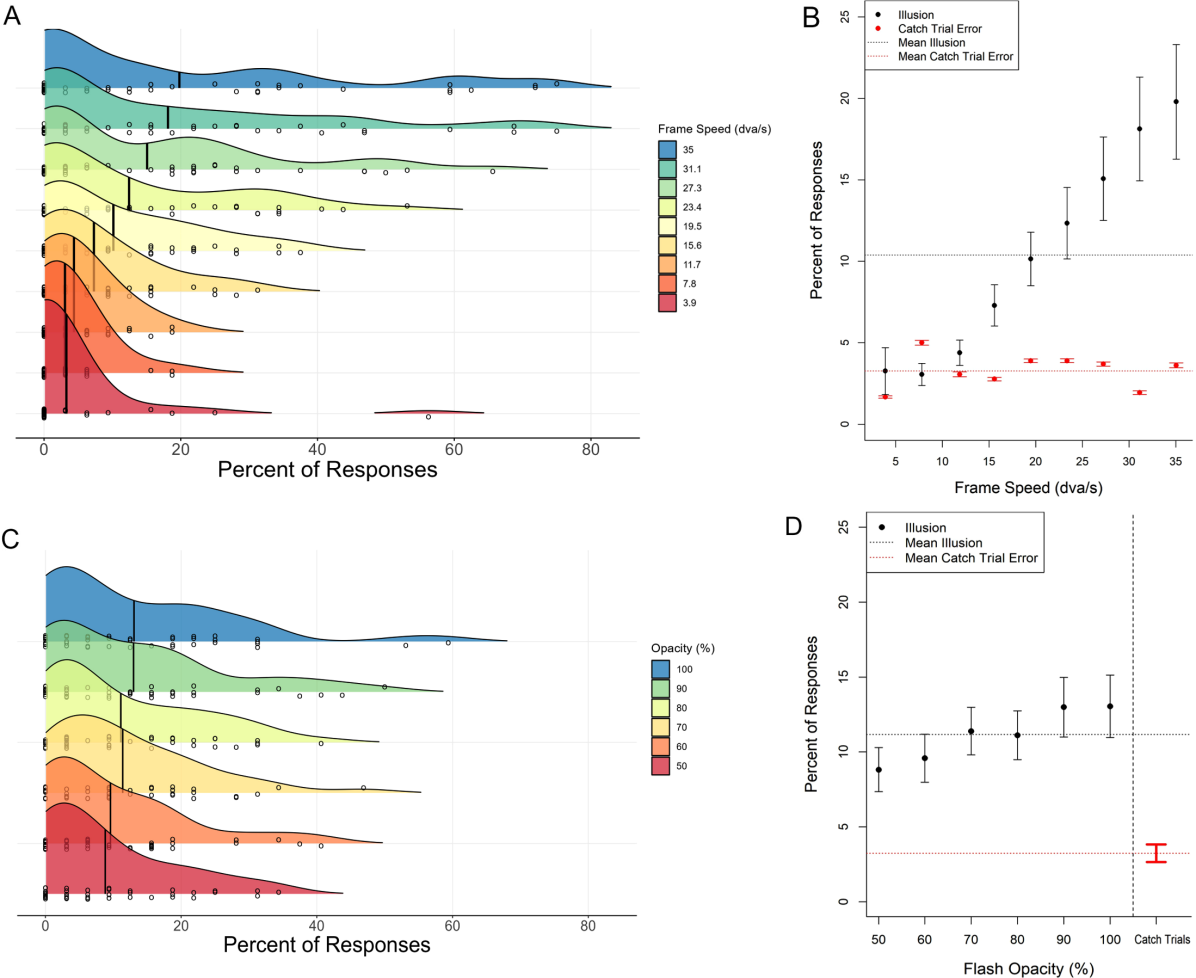
Frequency of reporting illusory splitting. (**A**) Vertical lines within the ridges indicate the group mean for that condition. Circles within the ridges indicate means for individual participants, with moderate vertical jitter so that values from overlapping individuals can be seen. (**B**) The percentage of experimental trials participants perceived the illusion compared to percentage of catch trial error in the speed condition. Error bars show ±1 SEM. Horizontal dashed lines show the mean across speed conditions. (**C**) and (**D**) show the same for the opacity condition. The ridges were created using the function geom_density_ridges() from the ggridges R package

The mean percentage of trials that participants perceived the illusion (i.e., reported seeing two dots) at the group level, as well as the distribution of mean percentages amongst individual participants, is shown for each frame speed in Figure 5A and for each flash opacity level in Figure 5C. The overall and within-manipulation mean rates of reporting illusion and catch-trial error can be seen for each frame speed in Figure 5B and for each flash opacity in Figure 5D.

In the frame speed condition (Experiment 1A), the overall percentage of trials that participants reported the illusion (*M* = 10.39, *SD* = 10.07) was significantly larger than the overall percentage of error in catch trials (*M* = 3.27, *SD* = 4.49), *t*(60.84) = 4.33, *p* < 0.001. Similarly, in the flash opacity condition (Experiment 1B), the overall percentage of trials that participants reported the illusion (*M* = 11.16, *SD* = 10.16) was significantly larger than the overall percentage of error in catch trials (*M* = 3.24, *SD* = 3.96), *t*(57.08) = 4.87, *p* < 0.001. This indicates that participants’ reports reflected perception of the illusion and not merely task error. This was corroborated by subjective reports from the debrief interviews, where 80% (*N* = 36) of participants said that they sometimes saw two dots during the experiment. Furthermore, a minority of participants (*N* = 8) indicated that they deliberately reported the illusion conservatively by defaulting to reporting one dot on trials where discrimination was difficult and reserving the two-dot response for trials in which they were certain that they saw two.

Linear regression analysis found that frame speed significantly positively predicted reporting the illusion, *R*^2^ = 0.15, *F*(1, 403) = 69.80, *p* < 0.001. This indicates that the split-stimulus effect becomes stronger with increasing speed. Flash opacity also significantly positively predicted reporting the illusion, *R*^2^ = 0.02, *F*(1, 268) = 4.63, *p* = 0.032, such that increasing the visibility of the target increased the chance that observers reported the illusion. However, we note that this effect was weak and only explained 2% of variance.

In studying a novel visual illusion in naïve participants, we are interested in the proportion of individuals who will spontaneously report seeing the illusion. Although group-level null hypothesis significant tests (NHST) can show whether average values are significantly different from one another, this analysis overlooks within-participant effects that are not captured in group level averages (see Ince, Paton, Kay, & Schyns, 2021). This fact is particularly pertinent to the present study given that, despite a significant group-level NHST, some of our participants reported never seeing the illusion, both in the task and in debrief interviews (*N* = 4), whereas others, as mentioned above, reported conservatively when discrimination was difficult (*N* = 8). As can be gleaned from Figures 5A and 5C, this resulted in positively skewed distributions of reporting with means that do not summarize the data well. Although our NHSTs have shown that participants overall reported perceiving the illusion significantly more than making errors in catch trials, a post hoc analysis was also performed to answer a different and complementary question: What is the estimated prevalence of individuals perceiving the illusion to a significant degree in the population?”

To address this, Ince et al. (2021) have recently developed a Bayesian technique that allows one to estimate an effect's prevalence in the population. The goal of this technique is explicitly to “quantify within-participant effects at the population level in a more meaningful way” than a reduction to binary conclusions of NHST inferences (Ince et al., 2021). Specifically, this method uses some test of significance to determine whether each individual reaches significance on a relevant metric. The number of participants reaching significance and the number of those failing to reach significance are then each added to 1 to attain the α and β values, respectively, of a beta distribution (i.e., α = 1 + n_significant_ and β = 1 + n_nonsignificant_). This beta distribution is then used to represent the population prevalence. The mode of the distribution represents the most probable estimate of population prevalence, termed the maximum a posteriori (*MAP*). The output of this Bayesian prevalence analysis also provides a natural interpretation of uncertainty, termed the highest posterior density interval (HPDI). The HPDI provides a range in which the true population prevalence can be found with a specified probability (Ince et al. recommend using specifically a 96% HPDI to emphasize the conceptual dissimilarity with 95% confidence intervals). This method allows clear quantitative claims about the probability of within-participant replication to be made (Ince, Kay, & Schyns, 2022). Hence, we used Bayesian prevalence analysis using a one-sided binomial test to determine whether within-participant rates of reporting the illusion, both within each condition level and overall across the entire condition, was significantly higher than the overall average error rate in catch trials. See the final paragraph of the methods section above for a link to the analysis code used.

**Figure 6.**
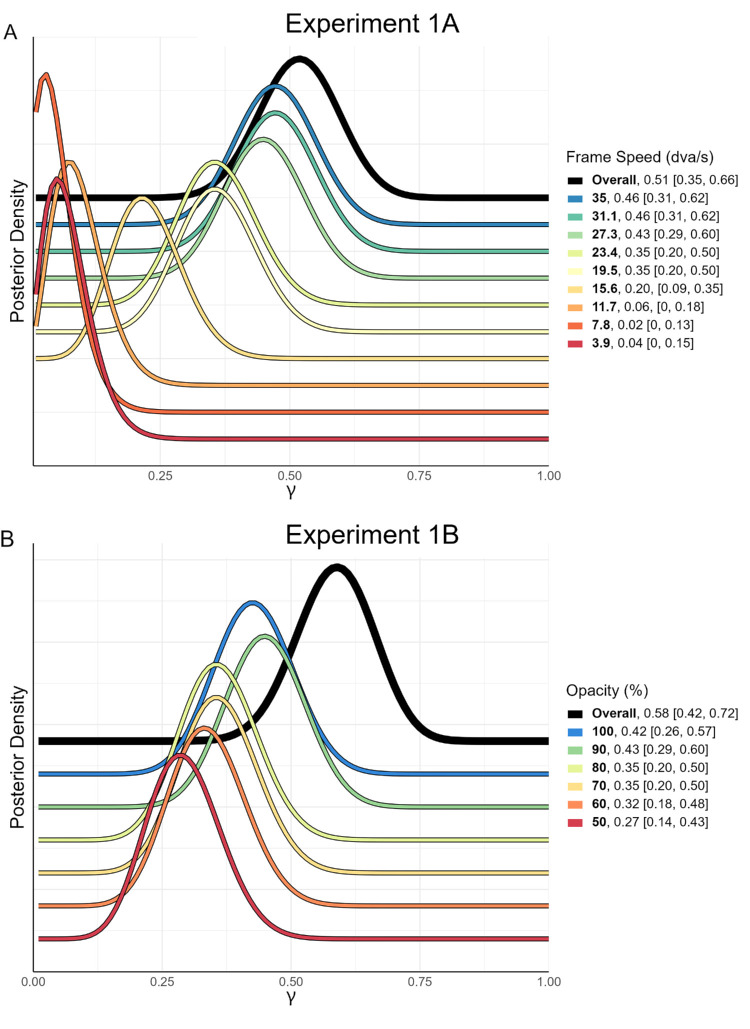
Bayesian posterior distributions of population prevalence. Experimental manipulation, as well as the maximum posterior density [96% HPDI] values are shown in the legend. The 96% HPDI values indicate that there is a 0.96 probability that true population prevalence will be above the lower bound and a 0.96 probability that true population prevalence will be below the upper. Lower-case gamma was chosen for the symbol denoting prevalence values on the x-axis in line with Ince et al. (2021)*.*

The results of the Bayesian prevalence analysis are shown in Figure 6. In the speed condition, 24 of the 45 participants showed overall significant reporting of the illusion. Therefore the overall maximum a posteriori estimate is γ = 0.51 (96% HPDI = [0.35, 0.66]). In other words, the posterior distribution indicates that 51% of naïve observers will report perceiving the illusion to a significant degree. The HPDI indicates that, given the data, the probability that the population prevalence of perceiving the illusion is greater than 35% is higher than 0.96. In the opacity condition, 27 of the 45 participants showed overall significant reporting of the illusion, with and overall *MAP* of γ = 0.58 (96% HPDI = [0.42, 0.72]. Note that a *MAP* of 0.02 in frame speed 7.8 dva/s indicates no participants reported seeing the illusion significantly more than mean error. This is simply the result of a beta distribution where α = (1 + 0) and β = (1 + *N*), or α = 1 and β = 46.

## Experiment 2

The purpose of Experiment 1 was to estimate the prevalence of the illusion in naïve observers under different speed and opacity conditions. As such, we recruited only naïve participants and deliberately avoided priming their percepts or reports during instruction. Having established that the illusion is spontaneously reported by the majority of participants, Experiment 2 further investigated the dependence of the illusion on two additional factors: the availability of selective visual attention, and the presence/absence of pre-reversal motion.

### Methods

#### Participants

Eighty-eight new participants were recruited using the same methods and prerequisites as in Experiment 1. Three participants were excluded: one due to a coding error that prevented data from being saved and two due to misunderstanding the instructions. The remaining 85 participants (64 females) were aged 18 to 49 years (*M* = 20.8, *SD* = 5.7).

In contrast to Experiment 1, participants in Experiment 2 were explicitly told that the experiment was investigating a novel visual illusion induced by motion in their periphery where a single red dot may appear as two dots adjacent to one another. Participants were given experiment instructions verbally with task instructions reproduced on the initial screen of the experiment.

#### Apparatus

Apparatus used in Experiment 2 was identical to that used in Experiment 1.

#### Stimuli

Stimuli in Experiment 2 were identical to those used in Experiment 1, with the exception that an additional, identical set of moving frames was added below fixation (see Supplementary Movie 2 and Supplementary Movie 3).

#### Experiment procedure

Experiment 2 involved two manipulations that were sequentially administered to the same group of participants. In Experiment 2A, we manipulated whether participants were able to attend to the position where the target would be flashed. Across three blocks, the red dot would either always flash (1) above fixation, (2) below fixation, or (3) flash randomly either above or below fixation. Block order was randomized, and a screen informing participants which block they were about to complete appeared before the first trial of each block.

In Experiment 2B, we investigated whether motion preceding the presentation of the target dot is required to induce the illusion. To do so, we presented stimulus sequences in which the frames only began moving immediately subsequent to the presentation of the dot; that is, there was no motion prior to the flash that could influence perception of the illusion.

Participants completed Experiments 2A and 2B in a randomized order. Each participant completed 900 trials in total: 600 trials in Experiment 2A and 300 trials in Experiment 2B. In Experiment 2A, the two blocks where the flash location was fully predictable comprised 150 trials each, whereas the block where location was randomized comprised 300 trials. In Experiment 2B the flash always appeared randomly either above or below fixation. Across both experiments the speed of the frames was also varied, moving at either 19.5°/s, 27.3°/s, or 35°/s on each trial. Breaks were built in every 20 trials. Unlike Experiment 1, catch trials were not included.

Although the purpose of Experiment 2B could have been realized with only one set of frames if the task was done in isolation, two sets of frames in Experiment 2B were included to keep the task as similar as possible to the task in Experiment 2A. Because the two tasks were done in a randomized order within the same session, this was done to eliminate any confounds to the effects of spatial attention measured in Experiment 2A that might appear if the task in Experiment 2B was performed first (e.g., by introducing a practice bias or an adaptation effect).

All participants completed 20 practice trials using stimuli as they appeared in Experiment 2A, with the red dot always appearing in the frames above fixation. As in Experiment 1, participants were debriefed and asked a series of questions about their experience after the experiment.

#### Trial procedure

**Figure 7.**
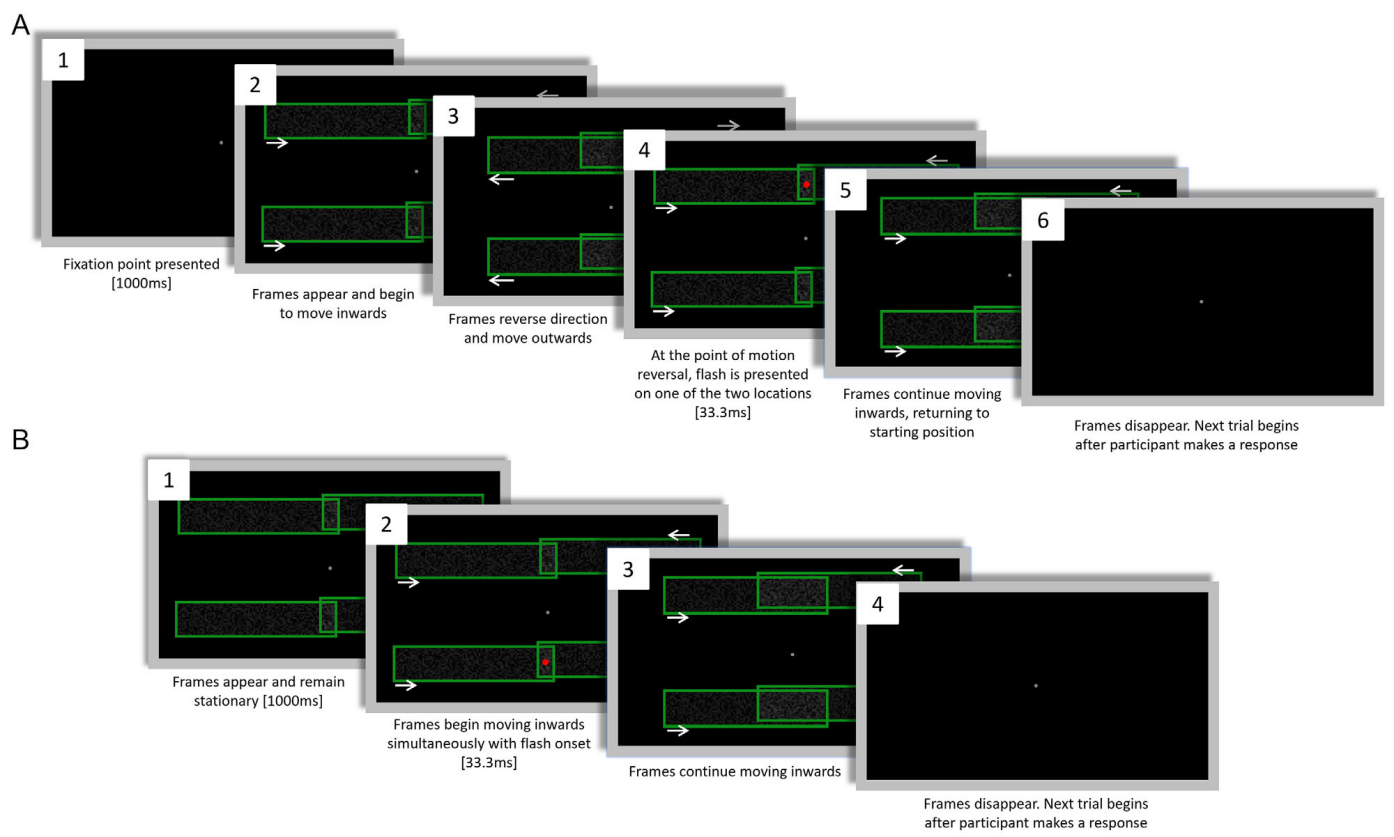
(**A**) Trial procedure for Experiment 2A and (**B**) for Experiment 2B. (**A**) Dashed white arrows in (2–4) indicate frame motion. (1) The fixation dot is presented on an otherwise blank screen. (2) The frames appear and begin moving toward one another. (3) The frames reverse direction and begin moving away from one another. (4) The frames reach the end of their trajectory and pause for 33.3 ms. During this window a red dot is randomly presented in one set of frames. (5) Simultaneous with flash appearance, the frames begin to move toward one another. (6) The frames disappear, and participants can make a response. (**B**) Trial procedure for Experiment 2B. Dashed white arrows in (2, 3) indicate frame motion. (1) The fixation dot and two sets of frames are presented for 1000 ms. (2) A red dot is randomly presented in one set of frames. Simultaneous with flash onset, the frames begin moving inward. (3) The frames continue moving inward. (4) The frames disappear, and participants can make a response. Figures not to scale.

Participants had the same three response options available to them as in Experiments 1A and 1B (0, 1, or 2 dots). Trial procedure for Experiment 2 was identical to Experiment 1 with the exception that each flash appeared only once per trial. In Experiment 2A, once the frames returned to the position that they appeared in, they disappeared. Such “single-shot” judgements were necessary to ensure that the location of the flash in randomized conditions could never be anticipated. The screen would remain blank until the participant made a response, causing the next trial to begin (see Figure 7A and Supplementary Movie 2).

In Experiment 2B, each trial started with both pairs of frames presented stationary for 1000 ms at the point where in the other experiments the frames would reverse direction. The frames began moving inward simultaneous with flash-onset. They continued to move inward until they disappeared at the same location as in Experiment 2A (See Figure 7B and Supplementary Movie 3).

### Results

The same analysis was performed as in Experiment 1 with one exception. Because there were no catch trials in Experiment 2, both the *t*-tests performed and the binomial tests used to estimate population prevalence in the Bayesian prevalence analysis compared rates of reporting the illusion against errors of omission (i.e., reporting 0 when 1 was presented) instead of against errors of commission (i.e., reporting 1 when 0 was presented). A one-way analysis of variance was run on the data from Experiment 2A to determine whether there was a significant difference in mean reporting of illusion among the three attention blocks. Subsequently, Bayes factors were calculated post hoc to estimate the relative likelihood that our manipulations of attention and frame speed affected the rate of reporting the illusion.

#### Experiment 2A

**Figure 8.**
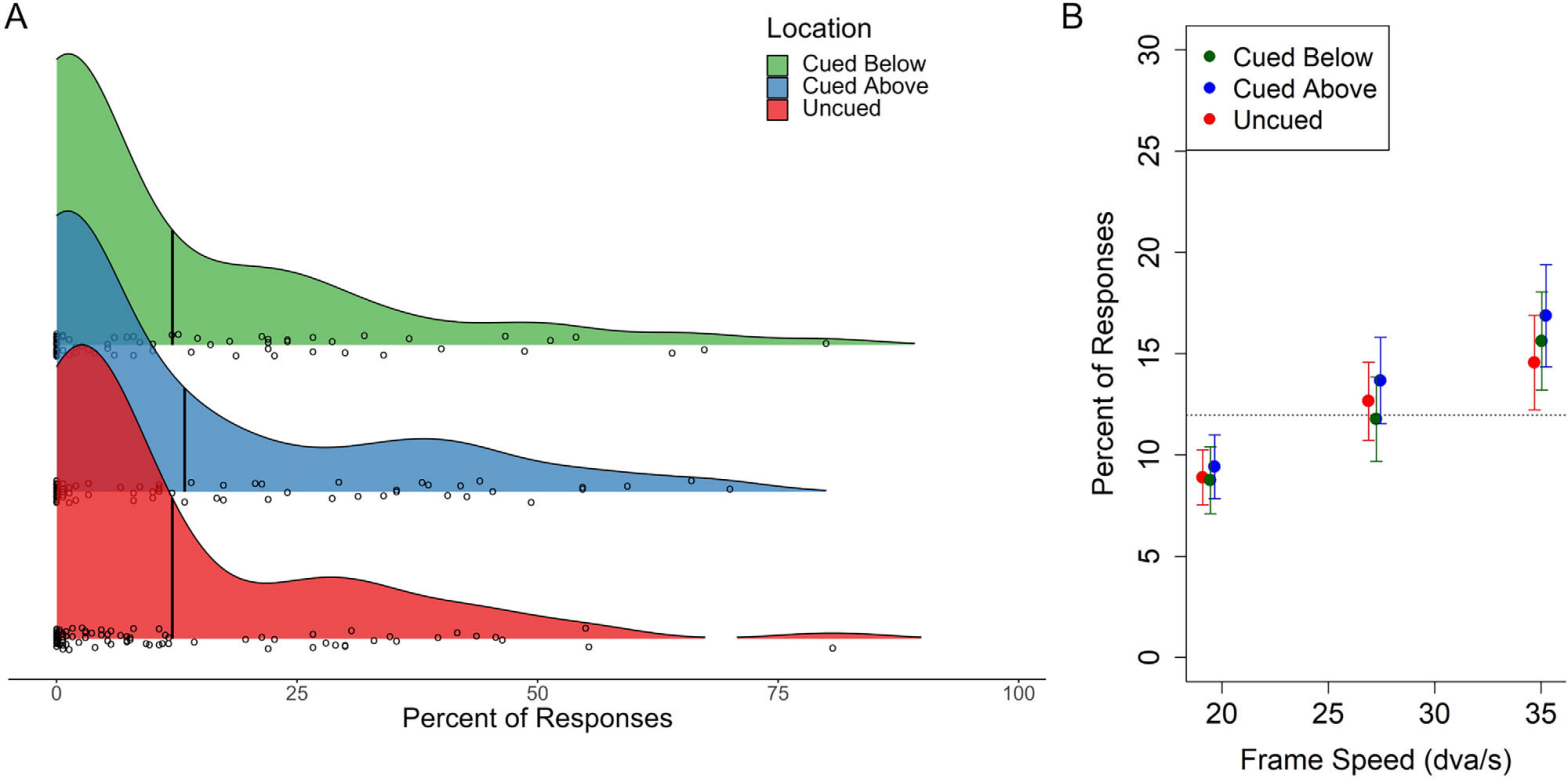
Percentage that individuals reported the illusion in Experiment 2A by cued attention. (**A**) The distribution of responses for each cueing condition. Vertical lines within the ridges indicate the group mean for that condition. Circles in the ridges indicate means for individual participants. They are jittered vertically so that values from overlapping individuals can be seen. (**B**) The mean percentage of experimental trials participants perceived the illusion for each speed and location of attention. Points and are jittered so that overlapping values can be seen. Error bars show ±1 SEM.

Figure 8A shows the mean percentage of trials that participants perceived the illusion at the group level as well the distribution of mean percentages amongst individual participants for each attention condition. Figure 8B shows means for each attention condition across frame speeds. A one-way analysis of variance found no significant effect of attention on mean illusion reporting, *F*(2, 252) = 0.26, *p* = 0.78. Furthermore, a Bayes factor indicated strong evidence for the absence of an effect of cueing spatial attention, *BF* = 0.05 (Lee & Wagenmakers, 2014).

**Figure 9.**
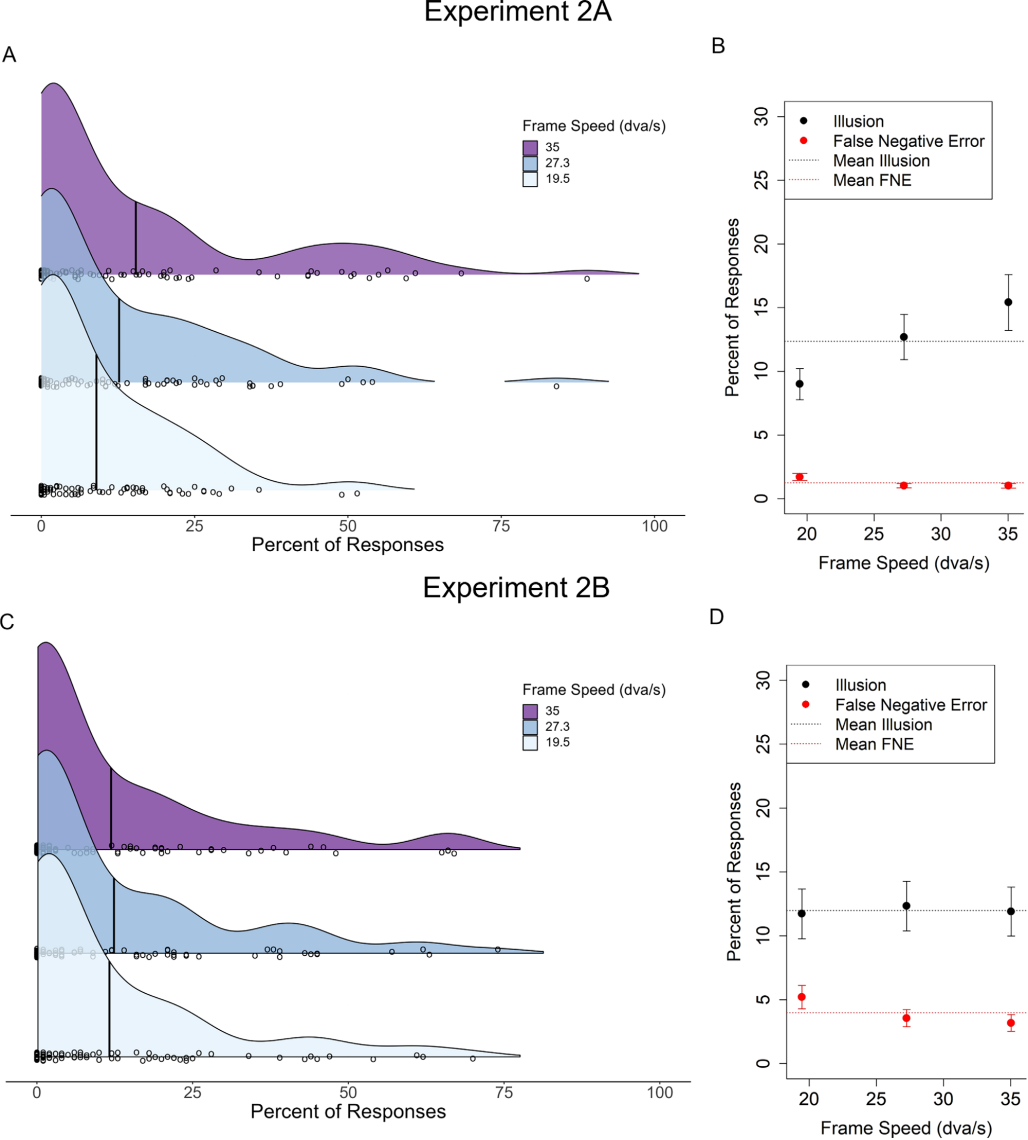
Distributions of the percentages that individuals reported in Experiment 2A and 2B by frame speed. (**A**) The distribution of responses for each frame speed collapsed across the attention conditions (Experiment 2A). Circles within the ridges indicate means for individual participants, with moderate vertical jitter so that values from overlapping individuals can be seen. (**B**) The mean percentage of experimental trials in which participants perceived the illusion compared to percentage of negative error for each speed. Error bars show ±1 SEM. (**C**) and (**D**) show the same for data from the motion onset condition (Experiment 2B).

Figure 9A shows the same data collapsed across attention conditions for each frame speed. Figure 9B shows the overall and within-manipulation mean rates of reporting the illusion and false negative errors. The overall mean percentage of trials that participants reported the illusion (*M* = 12.36, *SD* = 15.43) was significantly larger than the overall percentage of mean false-negative error (*M* = 1.24, *SD* = 1.77), *t*(86.21) = 6.60, *p* < 0.001. This indicates that participants’ reports reflected perception of the illusion as errors of commission (reporting the illusion) were significantly greater than errors of omission (task error). Linear regression analysis found that frame speed significantly positively predicted reporting of the illusion, *R*^2^ = 0.02, *F*(1, 253) = 6.53, *p* = 0.011.

#### Experiment 2B

In Experiment 2B, to investigate whether motion before the presentation of the dot was necessary to induce the illusion, the red dot was always flashed concurrently with the motion onset. The mean percentage of trials that participants perceived the illusion and the distribution of mean percentages among individual participants can be seen in Figure 9C.

Figure 9D shows the overall and within-manipulation mean rates of reporting the illusion and false-negative errors. As for preceding experiments, the overall percentage of trials that participants reported the illusion (*M* = 11.97, *SD* = 16.83) was significantly larger than the overall percentage of false-negative error (*M* = 4.13, *SD* = 6.74), *t*(110.24) = 3.99, *p* < 0.001. However, contrary to Experiments 1A and 2A, frame speed did not significantly predict reporting of the illusion in Experiment 2B, *R*^2^ < 0.001, *F*(1, 253) = 0.02, *p* = 0.92. This, despite the fact that the mean rate of reporting the illusion did not differ significantly between Experiments 2A and 2B, *t*(166.75) = 0.16, *p* = 0.88. A post hoc Bayes factor analysis indicated moderate evidence for the absence of an effect of frame speed in the flash-onset condition, *BF* = 0.14 (Lee & Wagenmakers, 2014).

#### Bayesian prevalence analysis (Experiments 2A & 2B)

As mentioned above, participants in Experiment 2 were told about the purpose of the experiment and nature of the illusory percept. This was because Experiment 1 already demonstrated that naïve participants spontaneously report the illusion, but also because it was thought knowledge of the illusion would minimize the issues with uncertainty participants expressed in Experiment 1, especially with the increased difficulty of a one-shot task. However, this meant that the frequency with which participants spontaneously reported the illusion was not the aim of Experiment 2. We note that given there was no false-positive, we had to use the false-negative error rate, preventing direct comparison with the estimates from Experiment 1. Nevertheless, we present MAP estimates here for completeness. For Experiment 2A, Bayesian prevalence analysis yielded an estimated *MAP* of 0.58 (96% HPDI = [0.46, 0.69]), and for Experiment 2B, the *MAP* was estimated to be 0.38 (96% HPDI = [0.27, 0.50]).

## General discussion

We report a novel visual illusion, the “split-stimulus effect”, in which participants report seeing a single object in two locations simultaneously. Through the lens of predictive processing, this effect indicates that two opposing perceptual predictions about an object can pierce consciousness concurrently. We found that naïve participants were significantly more likely to report seeing the illusion than make errors of commission (i.e., report seeing a dot when none was presented). We conservatively estimate the proportion of naïve individuals in the population who will spontaneously perceive the illusion at more than 50%.

We investigated several basic characteristics of the split-stimulus effect and compared its properties to both the frame effect and the flash-grab effect. First, we varied the speed of the inducing frames (Experiment 1A) and observed that increasing the frame speed significantly increased reports of illusory “splitting.” This suggests that the mechanism(s) underlying the split-stimulus effect may be different to those underlying the frame effect (which is speed insensitive), and similar those underlying the flash-grab effect (which is speed dependent). To determine how positional uncertainty affected perception of the illusion, we also manipulated the opacity of the flashed dots (Experiment 1B). Contrary to our expectations, increasing flash opacity was associated with a slight increase, rather than decrease, in the prevalence of the illusion.

In a follow-up experiment, we investigated the dependence of the illusion on the availability of spatial attention (Experiment 2A) and on the presence of motion preceding the flash (Experiment 2B). We observed that the illusion does not depend on pre-allocated spatial attention, with the illusion being equally prevalent in uncued and cued trials. Moreover, observers also reported the illusion when the target was flashed at the start of the motion sequence, without preceding stimulus motion, indicating that post-reversal motion is sufficient for generating the illusion (a “postdictive” effect, Eagleman & Sejnowski, 2000). Interestingly, however, although we replicated the dependence of the illusion on stimulus speed in Experiment 2A, when pre-reversal motion was removed (Experiment 2B) this dependence vanished.

### Estimate of population prevalence

Our analysis showed that, on average, participants reported perceiving the illusion significantly more often than they made errors in catch trials. When investigating a novel illusion, however, we are not just interested in comparing mean effects. We are also interested in investigating how often naïve people will report perceiving the illusion. Using a Bayesian prevalence analysis (Ince et al., 2021) and the data from Experiment 1, we estimated the population prevalence of the illusion at over 0.5, with a 96% HPDI lower bound of 0.35 for the speed condition and 0.42 for the opacity condition. As such, given the data, we would consider it highly likely that more than 35% of the naïve people would report the illusion significantly more than the average rate of spontaneous catch trial error, with the most likely estimate of true population prevalence over 50% (Ince et al., 2021). Given that some participants indicated in the debriefing interviews that they were conservative in reporting the illusion, this is likely to be a conservative estimate of the true population prevalence.

### Frame speed (Experiment 1A)

Findings from the frame speed manipulation show that increasing the speed of the background frames’ motion significantly increases the rate at which participants reported the illusion, with frame speed explaining ∼15% of the total variance in reporting. This speed-dependence suggests that the split-stimulus illusion may be more closely related to, and share an underlying mechanism with, the flash-grab effect than the frame effect (which is relatively speed insensitive).

What might this shared underlying mechanism be? Kwon et al. (2015) have argued that the brain possesses an internal model of motion dynamics, which it uses to infer the generative causes of incoming motion and position signals. They model this with a Kalman filter, which receives uncertain sensory measurements and produces state estimates (e.g., of position and velocity) by weighting current and past signals depending on their relative uncertainty (for a related model see Grush, 2005). The upshot is that when uncertainty about current sensory input is high (e.g., during peripheral viewing), estimates of objects’ states are more heavily influenced by past signals and internal predictions. Local motion signals are also more likely to be misattributed, leading to a bias in the predicted, and thus ultimately perceived, position of an object. From this perspective, the common speed-dependence of the split-stimulus and flash-grab effects may be due to the shared involvement of such an inference mechanism, with faster speeds leading to greater biases in position estimates.

Interestingly, when pre-flash motion was removed (i.e. when the flash was presented at the onset of motion) we found that this speed-dependence disappeared (Experiment 2). This suggests that either the aforementioned inference process is disrupted (i.e. that it takes a while to develop speed sensitivity) and/or that additional (speed-insensitive) mechanisms are involved in generating the illusion, which can be modulated by pre-flash motion. To this latter point, recent research suggests that phenomenologically similar illusions may arise from multiple mechanisms, some shared and some unique (Cottier, Turner, Holcombe, & Hogendoorn, 2023). The absence of speed-dependence in Experiment 2B in some ways mirrors the characteristic speed insensitivity of the frame effect, potentially indicating a common mechanism. Özkan et al. (2021) found that the degree of perceived position shift was predicted not by frame speed but by the length of the path the frame travelled between motion reversals. Future study of the split-stimulus illusion could therefore investigate whether systematically varying the path length of the inducing frames affects perception of the current illusion. More generally, future studies could adopt an individual differences approach (e.g., Cottier et al., 2023) to directly examine the degree to which the split-stimulus effect correlates, and thus shares mechanisms with, the frame effect, flash-grab effect and other MPIs.

An alternative avenue for future studies regards the features of the frames themselves. The characteristics of the frames were chosen based on what subjectively maximized the strength of the illusion during piloting. We conjecture that the inclusion of both frame outlines and luminance noise provides converging evidence that there are two gestalts moving transparently in opposing directions, reducing evidence for a competing interpretation (e.g., without luminance noise, the two frames may look like a single abstract shifting mesh) and thereby boosting the illusion. Similarly, making the frames wide meant that they were overlapping for the entirety of their traversal. This may have boosted the illusion as it meant there was always motion information in the location participants attended to, the location where the flashed dots appeared. Because Özkan et al. (2021) found that the frame effect requires that the length of the frames’ traversals is less than twice their width, we assumed making the frames wide would help increase the likelihood of perceiving the split-stimulus effect, or at the very least would not harm the likelihood. However, because the frames were never varied in any way, these explanations remain conjecture.

Importantly, this interpretive ambiguity was not an issue for the frame effect (Özkan et al., 2021) because participants had only to track the motion trajectory of a single object. The addition of luminance noise may end up being innocuous; however, the change may contribute to engaging an entirely separate mechanism than that which produces the Frame Effect or engaging a mechanism that contributes to the flash-grab effect. Given the conflicting results regarding the effects of frame speed in Experiments 1A and 2B, future studies may examine the relative importance of these features (e.g., frames vs. noise) in generating the present illusion. This would help to clarify the taxonomic relationship between this and other potentially related illusions (e.g., the flash grab and frame effects).

### Flash opacity (Experiment 1B)

Contrary to our hypothesis, decreasing the opacity of the flashed dot slightly decreased, rather than increased rates of reporting the illusion. Based on previous research, we expected that as the opacity of the flash decreased from 100% to 50%, the flash's positional uncertainty would increase (Kanai et al., 2004; Sceniak, Ringach, Hawken, & Shapley, 1999), leading to greater illusory shifts. One potential reason why we found the opposite effect is that manipulating the opacity of flashes did not vary positional uncertainty because of the eccentricity at which the flashes were presented. Specifically, Kanai et al. (2004) found an effect of flash contrast on the size of a perceived position shift for stimuli that were presented relatively close to fovea. However, segmentation of motion signals is highly sensitive to contrast in luminance signals at lower eccentricities but not so at higher eccentricities (Murakami & Shimojo, 1993; Ramachandran, 1987). By decreasing the flash opacity, then, we may have manipulated a signal that does not have large effects on the segmentation of motion at the eccentricity the stimuli were presented. Furthermore, reducing opacity at a relatively high eccentricity may have in fact decreased the flash's detectability, a confound that the current paradigm is not designed to disentangle. Future studies may instead be able to investigate the effects of positional uncertainty by a paradigm in which the eccentricity of stimuli presentation is varied, or in which the border of a stimulus is blurred (see Kwon et al., 2015). Ultimately, the small positive effect of flash opacity on reports of perceiving the illusion remains somewhat puzzling, and warrants further investigation.

Relatedly, it is interesting to note that the mean rates of reporting the illusion were more or less constant across Experiments 1 and 2. This is somewhat surprising, as participants in Experiment 1 could view many repetitions of the stimulus in a single trial, and so could accumulate sensory evidence over time. Nevertheless, they ended up reporting the illusion with more or less the same frequency as participants who only saw a single instance of the stimulus (Experiment 2). This may suggest that even with repeated exposure to the stimulus, the illusory percept, if perceived, can stabilize (similar to how ambiguous images stabilize on one interpretation). However, it's important to note that differences between these experiments (e.g., participants in Experiment 2 were non-naive, and performing a more difficult task) precludes direct comparison of their results and thus limits strong conclusions from being drawn.

### Spatial attention (Experiment 2A)

Our findings from Experiment 2A show that the availability of spatial attention did not affect rates of reporting the illusion. Specifically, whether or not participants could anticipate the precise location of the flash did not affect the likelihood of them seeing illusory splitting. This finding is curious, because the flash-grab effect and other MPIs such as the “twinkle goes” effect (Nakayama & Holcombe, 2021) have been shown to be significantly affected by attention (e.g., Cavanagh & Anstis, 2013; Coffey et al., 2019). One reason why we may not have observed an effect is because the specific attention manipulation we used was relatively modest. Given that there were only two possible stimulus appearance positions, participants may have been able to divide their attention across locations. However, Nakayama and Holcombe (2021) used similar attention manipulation and observed a clear attention effect for the twinkle-goes illusion. As such the present lack of attention dependence is a factor that sets the split-stimulus effect apart from the twinkle goes effect, and potentially related illusions like the flash-grab effect. Looking forward, future studies may consider using an invalid cue condition in addition to valid and no-cue conditions to more strongly manipulate attention.

Although the present study examined the effects of spatial attention, we did not explore the effects of selective attention. In a study examining properties of the frame effect, Cavanagh et al. (2022) found that shifting attention between two simultaneously available frames resulted in a position shift relative to the attended frame. Because it is possible the split-stimulus effect is a result of simultaneous splitting attention between the motion trajectories of two frames, future studies could examine whether instruction to attend to one of the two frames in the split-stimulus effect results in the percept of a single dot shifted in the direction of the attended frame.

### Preceding motion (Experiment 2B)

Our findings from Experiment 2B show that the Split Stimulus Effect occurs even in the absence of pre-flash motion. The same is true for the flash-grab effect (and indeed the flash lag effect, Eagleman & Sejnowski, 2000) where mislocalization is driven primarily by motion after, rather than before the flash (Anstis & Cavanagh, 2017; Blom et al., 2019; Cavanagh & Anstis, 2013; Takao et al., 2022; van Heusden et al., 2019). As noted earlier, when pre-flash motion was removed, the illusion became speed-insensitive. This is inconsistent with the findings of Blom et al. (2019), who investigated the relative contributions of motion before and after the flash in the flash grab effect. In contrast to our findings, they found that the speed of pre-flash motion did not affect perceived position. Effects were found only when post-flash motion speed also varied—the opposite pattern of our current findings. However, modelling evidence indicates that having motion presented both before and after the flash, as in Blom et al., may engage a different number and/ or kind of mechanisms then when motion is presented only after the flash, as in Experiment 2B (Takao et al., 2022). As such, our ability to directly compare these results is somewhat limited.

Nevertheless, despite their differences, these findings collectively demonstrate that both pre- and post-flash motion can affect perceived position. Given that post-flash motion is sufficient for driving the effect, the Kalman filter mechanism discussed above is more appropriately conceptualized as a Kalman “smoother,” in which signals arriving later in time can be used to refined earlier state estimates (Rao, Eagleman, & Sejnowski, 2001; Grush, 2005). Within this framework, our results suggest that the split-stimulus effect may arise, in part, from a mechanism which is naturally speed insensitive but which can modulated by pre-flash motion (hence, why the average rate remained unchanged in Experiment 2B). Alternatively, the mechanism may be speed-dependent but simply take a while to “come online” and incorporate velocity information. To differentiate between these possibilities, and more thoroughly characterize the speed-dependence of this illusion, future studies could independently vary the speed of pre- and post-flash motion (as in Blom et al. 2019).

### Limitations

#### Response measures

One could also interpret the unexpected null effect of post-flash motion velocity as being due to differences in task procedure — the Flash-Grab Effect typically estimated using a continuous measure of the degree of perceived shift (e.g., Blom et al., 2019; Cavanagh & Anstis, 2013; Coffey et al., 2019). For our experiments, participants made categorical responses, simply indicating how many dots were perceived per trial. Reporting “2” therefore served as a proxy for a degree of position shift sufficient to induce splitting. It cannot be conclusively deduced, however, whether the degree of position shift sufficient to produce the illusion is the product of a continuous process that is amplified by increased frame speed or is the product of a binary process that is more likely to get engaged at increased frame speeds. (Interestingly, in the debriefing interviews some participants indicated that they saw an “oblong” or “rectangular” stimulus. This suggests the operative mechanism is the former, which can sometimes lead to partial/incomplete separation of the two predicted flash locations). Similarly, it may be that post-flash motion velocity does influence the degree of positional shifts in the split-stimulus effect but in a manner that was not captured with our categorical measure. Alternatively, given pointing measures require the engagement of certain mechanisms such as attention-shifting and short-term memory, it may be that the resulting data are more liable to be affected by motion velocity (Kerzel, 2010).

A continuous measure, for instance one where participants manually indicate the size of the gap between the two dots, may provide a more direct measure of the degree of position shift. However, we did not adopt such a continuous measure in the current study primarily due to concerns it would encourage unintentional saccades away from fixation, and naïve participants would consequently discover the nature of the task. Having now demonstrated the illusion in a naïve sample, a continuous measure could offer a fruitful method of investigating how different parameters affect the degree of splitting, particularly with respect to the interaction between motion speed and the timing of motion onset.

#### Detection versus discrimination

To quantitatively demonstrate that naïve participants saw the illusion, we compared the rate individuals reported perceiving the illusion in experimental trials to the rate they made errors in catch trials. An astute critic, however, might notice that we are comparing the error rates of two qualitatively different tasks: a detection task (presence versus absence of dots) and, if something is detected, a discrimination task (one dot vs. two dots). We are making the tacit assumption that the error rates in these two tasks are approximately the same, perhaps erroneously. This could mean that participants weren't reporting the illusion at a rate significantly more than the rate of making expected spontaneous errors but merely making more discrimination errors compared to detection errors.

Skepticism about the rate participants perceived the illusion can be assuaged with a few considerations. First, conceptually speaking both making an error in a catch trial and reporting seeing the illusion are a “false positive.” Although measuring catch trial errors is not a perfect control, as a measure of how often participants spontaneously reported seeing something additional to what was presented, it is still a relevant comparison. Second, participants could view each trial for as long as they required before responding. That is, they could accrue as much evidence as necessary to reduce uncertainty in their decision. It seems unlikely, then, that the higher average rate of reporting the illusion would be driven by, for example, higher difficulty of the discrimination task compared to the detection task due to differences in evidence-accrual thresholds.

Still, perhaps the average rates of reporting the illusion could be explained by subject-expectancy bias. Two dots were never flashed in the experiment; participants may have reported perceiving two dots because they felt they were expected to use all the responses provided. Although participants were instructed that it was OK if they used some responses more than others, the more striking piece of evidence to assuage this concern is rather simple: during debrief interviews a large majority of participants confirmed that they saw the illusion in the way expected over the course of the experiment. Indeed, some participants explicitly indicated that they reported the illusion conservatively, reserving the two-dot response for trials in which they were certain they saw two, suggesting underreporting may be a bigger concern than overreporting. Moreover, the fact that these participants reserved reporting the illusion for when they were certain that they perceived two dots establishes that these participants *were* certain about the illusory percept at least some of the time. These subjective reports are powerful evidence that our interpretation of the results does not confuse signal with noise.

### The split-stimulus effect, predictive processing, and hyperpriors

**Figure 10.**
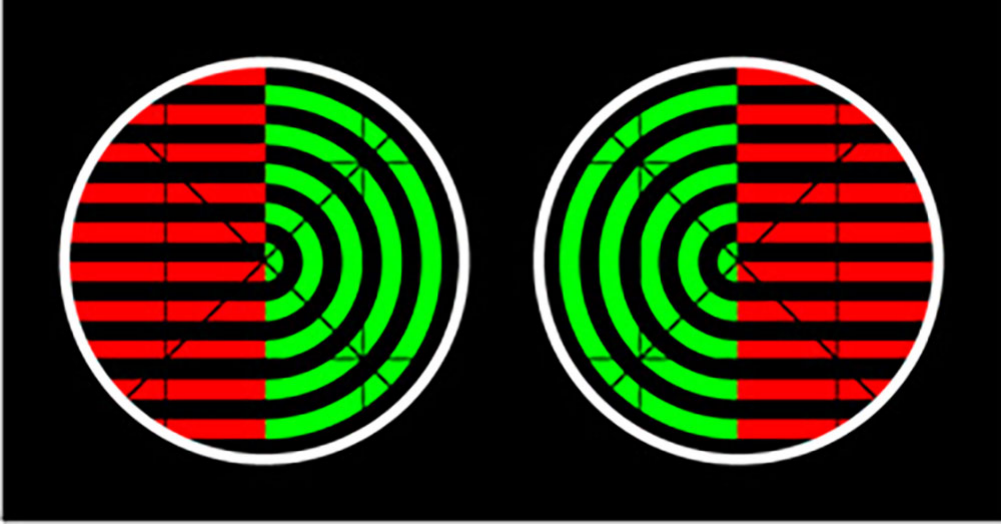
Stereoscopic stimuli for binocular rivalry from Diaz-Caneja (1928). Although each stimulus shows half of each pattern, rivalry occurred between the whole patterns. Adapted from “On Binocular Alternation” by D. Alais, R. P. O'Shea, C. Mesana-Alais, and I. G. Wilson, 2000, *Perception*, 29, p. 1443. Copyright © 2000 by SAGE Publications. Reprinted by Permission of Sage Publications.

To understand the broader significance of the split-stimulus effect, it is helpful to contrast it with binocular rivalry (see Alais, 2012 for review). Binocular rivalry is a multistability phenomenon where the brain must resolve competition between interpretations of visual stimuli presented to each eye in isolation. Diaz-Caneja (1928) used a binocular rivalry paradigm to present half of two different patterns to each eye, shown in Figure 10, Diaz-Caneja found that rivalry occurred between percepts containing each of the whole patterns. This puzzling result can be explained relatively easily in the Bayesian predictive processing framework: Although there is a high likelihood that the sensory evidence is being caused by sudden disjoints in the two patterns (i.e., the veridical stimuli), the prior probability of this hypothesis is exceedingly low. Instead, there is a high prior probability that the percept is a coherent pattern, and this prior expectation biases perceptual processing, influencing the abductive process (i.e., what is considered the “best” explanation; Hohwy, 2013).

In the above example, rivalry occurs between whole patterns rather than the veridical stimuli because of prior probabilities that bias visual interpretation toward the two percepts that are the most coherent. In turn, the cause of perceptual alternation between these two coherent percepts can be understood in Bayesian terms by appealing to “hyperpriors”—prior probabilities about prior probabilities—that constrain the kind of hypotheses that the brain's models are able to generate (Hohwy, Roepstorff, & Friston, 2008). These constraints are necessary for the brain to narrow down possible hypotheses and carry out its function of inferring the latent causes of its sensory inputs (Tenenbaum, Kemp, Griffiths, & Goodman, 2011). That is, they are necessary for experience itself to be coherent. The specific hyperprior that binocular rivalry demonstrates is that multiple objects cannot be located in the same spatiotemporal position. The two equally likely conflicting hypotheses generated in binocular rivalry are therefore mutually exclusive, meaning at every time point only one of the perceptual inferences can be represented in consciousness and the other must be inhibited.

In contrast, the split-stimulus illusion demonstrates a novel resolution to this situation of conflicting perceptual hypotheses. When the illusion is induced, two equally likely hypotheses are made about a single object. However, because of the illusory shifts in position, the otherwise conflicting hypotheses are not mutually exclusive—by inducing shifts in the flashed dot's predicted spatial location, the illusory percept bypasses the constraint of the spatiotemporal hyperprior. That is, from the perceptual system's point of view (so to speak), simultaneous perceptual predictions about different spatial locations simply means that two different objects are present. This means the two conflicting predictions about the flash's position can pierce consciousness simultaneously. As far as we know, this experiment is the first to show that such a phenomenon is possible, providing new insight into the mechanisms of hierarchical predictive processing including how the mind structures visual experience into space and time itself.

### Individual differences

Interestingly, we observed large individual differences in the rate at which participants reported seeing the illusion. Although some participants reported seeing the illusion in the majority of experimental trials, the overall mean rate was relatively low (∼12% of trials). As we have already noted, this estimate may have been deflated by conservative reporting strategies or by the use of a relatively coarse discrete reporting criteria. Nevertheless, it is clear that people do not experience the illusion 100% of the time. This may be due to nuisance factors (distraction, fatigue, breaking fixation, etc.), but it may also be indicative of something more interesting. Specifically, it may indicate the existence of a threshold that determines whether a perceptual prediction is consciously rendered. Only when two competing predictions are sufficiently likely will they both be concurrently experienced. From this viewpoint, individual differences may arise, in part, because of differences in the specifics of this threshold across participants. To further examine this idea it will be important to correlate individual differences in illusion reporting across multiple session, to determine their stability. Neural measures may also be used to attempt to quantify the amount of “neural evidence” for each perceptual prediction and to examine whether their relative magnitudes relate to their likelihood of being perceived.

## Conclusions

In the naïve empiricist theory of John Locke, the brain is a passive audience to the rich representation of the world contained in the signals of the sensory organs. In light of research into the perceptual misrepresentations that characterize perceptual illusions, this perspective must be replaced with one where the mind has an active role in generating perceptual content, with representations in consciousness understood to be the result of the brain actively predicting incoming sensory signals. The present study investigated a novel visual illusion in which symmetrical transparent background motion can cause a single flashed stimulus to be perceived to “split,” appearing in two locations simultaneously. In addition to quantitatively demonstrating the phenomenon in naïve subjects, we characterized the illusion's dependence on a number of relevant parameters and compare the effect to other motion-position illusions, including the frame effect and the flash grab effect. More fundamentally, the present study illustrates a novel consequence of the predictive processing paradigm, where motion not only causes an object's position to be misrepresented, but where two opposing misrepresentations about a single object's position can both pierce consciousness simultaneously (on the condition that the representations do not share a spatiotemporal location). In doing so, the split-stimulus effect highlights an important hyperprior regarding the incorporation and organization of sensory inputs in perceptual space over time.

## Supplementary Material

Supplement 1

Supplement 2

Supplement 3
